# Comparative study of quantitative identification methods for peri-urban areas based on a multi-indicator system

**DOI:** 10.1038/s41598-024-80848-7

**Published:** 2024-11-27

**Authors:** Zhen Shi, Manshu Liu, Ying Wang, Krisztina Filepné Kovács

**Affiliations:** 1 Department of Landscape Planning and Regional Development, Hungarian University of Agriculture and Life Sciences, Villányi Út 29-43, 1118 Budapest, Hungary; 2 College of Architecture and Engineering, Zhengzhou University of Industrial Technology, No. 16 Xueyuan Road, Xinzheng, 45100 China

**Keywords:** Peri-urban areas, Threshold method, Breakpoint clustering, Multilayer perceptron, Urban ecology, Sustainability

## Abstract

As peri-urbanisation becomes widespread, quantitative methods for identifying peri-urban areas (PUAs) are increasingly abundant. This study aims to quantitatively compare the differences in the results obtained from multiple PUA identification methods within the same study area and to analyse the spatial patterns of the PUAs. The Threshold Method, Breakpoint Clustering, and Multilayer Perceptron were chosen to compare the identification results of PUAs in Zhengzhou City, China. The results show that the Threshold method identified the most PUAs but with the lowest accuracy. The accuracy of the Breakpoint Clustering was slightly lower than that of the Multilayer Perceptron. Regarding spatial layout, these identification results all show that the PUAs in the northeastern plains of Zhengzhou are more numerous than in the southwestern mountainous regions. Moreover, PUAs can generally be categorised into ring-shaped, belt-shaped, and patch-shaped forms. The spatial evolution of PUAs is influenced by urban expansion, geographical factors, and urban planning, and it can also provide timely reflections on urban development dynamics. These findings can provide valuable references for future research in selecting PUA identification methods, promoting in-depth longitudinal studies and cross-regional research, and enhancing the attention given to PUAs in urban planning and policy-making.

## Introduction

Peri-urban areas (PUAs) are typically defined as transitional zones emerging at the fringes of large urban regions and rural areas where non-agricultural industries are significant^1,2^. These areas are characterised by a mix of urban and rural landscapes and functions, demonstrating a high degree of hybridity^3,4^. With the advance of global urbanisation, the phenomenon of peri-urbanisation has also emerged in various regions around the world. This trend is typically accompanied by shifts in landscape patterns and socioeconomic changes, leading to the emergence of numerous theoretical and empirical studies on PUAs^5^. After studying the phenomenon of peri-urbanisation in East Asia, Webster (2002) argued that peri-urbanisation is a highly dynamic process^1^. Amirinejad et al. (2018) discussed the ambiguities in the definition, characteristics, types, and policy formulation and implementation of PUAs in Australia^6^. Adam (2020) explored conflicts in Ethiopia’s PUAs from a political economy perspective, finding that the primary competing interest groups are the state, the private sector, and local communities^7^. Seifollahi-Aghmiuni et al. (2022) reviewed land degradation in PUAs of Southern Europe, suggesting that PUAs serve as a new “laboratory” for studying the intrinsic relationship between humans and nature, as well as a socio-environmental system adapting to intense socioeconomic transitions^8^. Chettry (2022) analysed the evolution of PUAs in the Thiruvananthapuram Urban Agglomeration in India and found that discontinuous, low-density development dominated the types of expansion in the PUAs^9^. Tiwari and Vajpeyi (2023) used bibliometric analysis to examine global literature on PUAs. The study found that key research hotspots in PUA studies primarily focus on land use and land cover change, urbanisation processes, and their environmental impacts^10^. In these studies from around the world, PUAs have shown their significant roles in optimising urban development, promoting sustainable land use, and improving ecological environments, which is why they have become a focal area of interest within the academic community.

In the study of PUAs, defining the boundaries of PUAs is often the primary step. Precise demarcation of PUA boundaries not only clarifies the scope of research but also provides a crucial basis for analysing spatial evolution patterns, changes in land use and landscape configurations. However, the academic community has long been engaged in ongoing debates over the identification of PUAs, and to date, there has yet to be a consensus on a unified theory or methodology. Through a review of the literature, we found that the identification of PUAs involves two stages: qualitative identification and quantitative identification^11^. Early studies commonly relied on experiential identification, where the extent of PUAs was defined as several kilometres beyond built-up areas, varying by region^12–15^. Subsequently, qualitative identifications gradually gave way to quantitative methods, marking a significant shift in research methodologies. Among quantitative methods, the Threshold method has emerged early and is widely applied^9,16,17^. Subsequently, emerging methods such as the breakpoint method^18,19^ and spatial clustering^20–23^ have been applied in many study areas. Notably, with technological advancements, machine learning techniques have also been introduced into PUA research, demonstrating immense potential for enhancing identification precision^24^.

The selection of indicators and the determination of research units are also essential aspects in identifying PUAs^25^. Using a single indicator can yield results more quickly, but the accuracy of the indicator data significantly impacts the outcomes. In contrast, constructing a multi-indicator system with multiple data sources can mitigate this effect by reducing the influence of any single data point on the results. Consequently, multi-indicator systems are more widely adopted in research^20,21^. In past studies, common indicators have typically been derived from satellite imagery or statistical data, including land use, population, and economic metrics. These studies assume that PUAs exhibit values for these indicators that lie between those of urban and rural areas, thereby distinguishing them^20,21,26^. In terms of units of study, administrative boundaries, due to their ease of access and compatibility with statistical data, have been favoured by many researchers^27,28^. Grid units have become increasingly popular for their uniform and adjustable scale^29–32^.

Although there are some studies on the selection of PUA identification methods, most of them are in the form of literature reviews. Cattivelli (2021) explored the methods for identifying PUAs in Europe in recent years and found that the most widely used approaches are those based on demographic and socioeconomic variables^25^. Mortoja et al. (2020) and Sahana et al. (2023) both emphasised the importance of identifying PUA boundaries for formulating protection and management policies. Through a review of the relevant literature, they concluded that due to the regional characteristics of PUAs, there is no universal method for delineating them^33,34^. These literature reviews provide us with valuable insights when selecting identification methods. However, the presence of various variables (such as differences in study areas, indicators, and research units) results in a lack of straightforward comparative outcomes when reviewing these methods. For researchers, conducting practical comparisons and screenings of multiple identification methods in a specific study area before determining the appropriate method is a necessary step. This process will help select the most suitable approach for the research area. However, current research findings indicate that there is still a gap in studies comparing the accuracy and applicability of different PUA identification methods within the same research area.

**Fig. 1:**
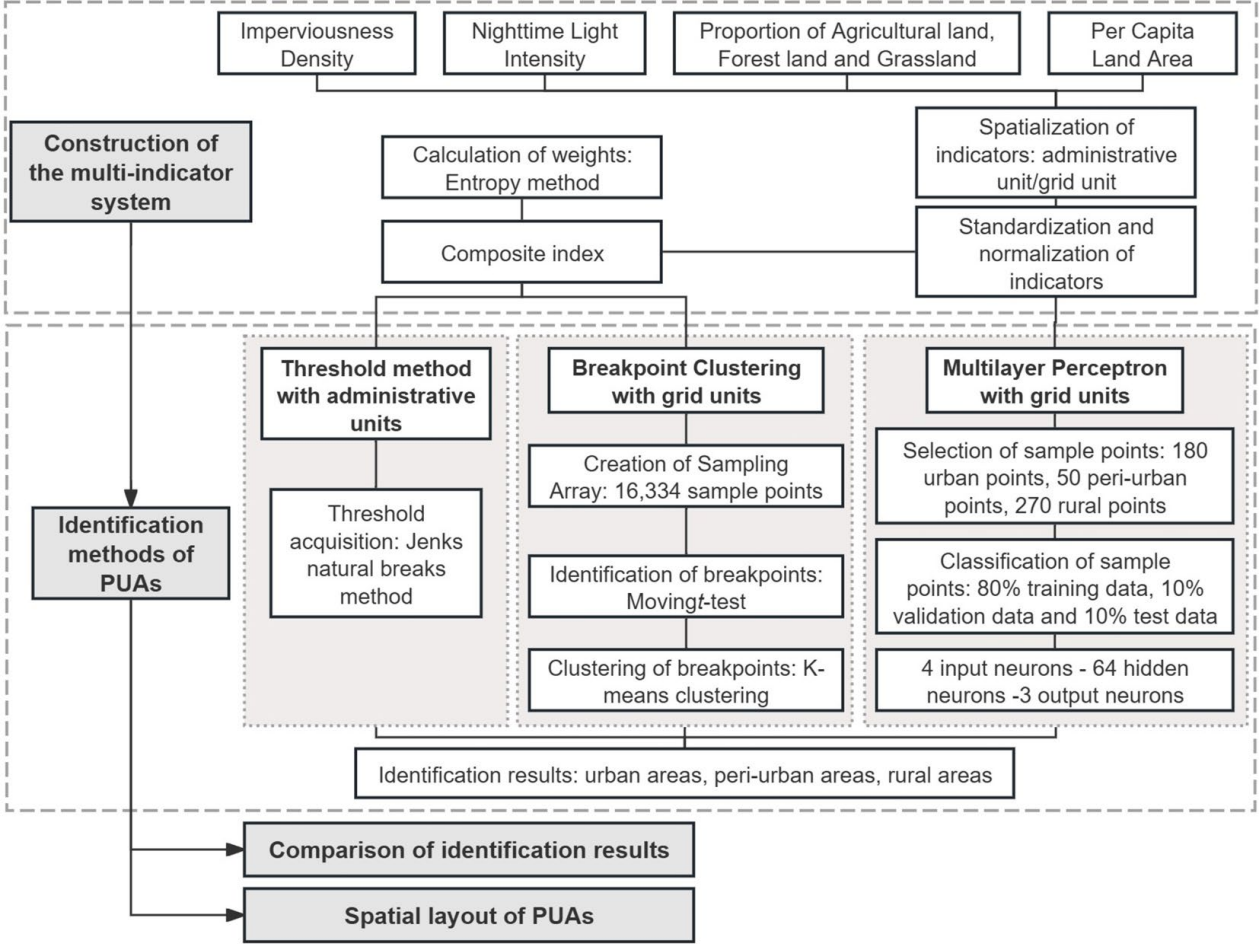
Technical framework for comparing quantitative identification methods of PUAs.

The purpose of this study is to quantitatively compare the differences in the results obtained from multiple PUA identification methods within the same study area and to analyse the spatial patterns of the PUAs. The indicator system used in this study includes Imperviousness Density, Nighttime Light Intensity, Proportion of Agricultural land, Forest land and Grassland, and Per Capita Land Area. The methods for comparison include the Threshold method, the Breakpoint Clustering, and the Multilayer Perceptron (Fig. 1). Additionally, we evaluated these methods in terms of their research units, operations, and applications, hoping to assist researchers and policymakers in assessing the applicability of these methods in different regions and contexts. In this study, we selected Zhengzhou, an emerging megacity in China, as the case study. Megacities are measured by population size, and they also represent cities with a relatively high level of economic development that have an intense radiation and driving effect on surrounding areas^35^. The peri-urbanisation phenomenon in these megacities is often in a distinctly characteristic stage, making them suitable for the identification study of PUAs^36^. In summary, the research questions addressed by this study are: How do the Threshold method, Breakpoint Clustering method, and Multilayer Perceptron perform in identifying PUAs within the same study area and using the same multi-indicator system? What spatial patterns are exhibited by the PUAs revealed in the identification results?

## Materials and methods

### Study area

**Fig. 2:**
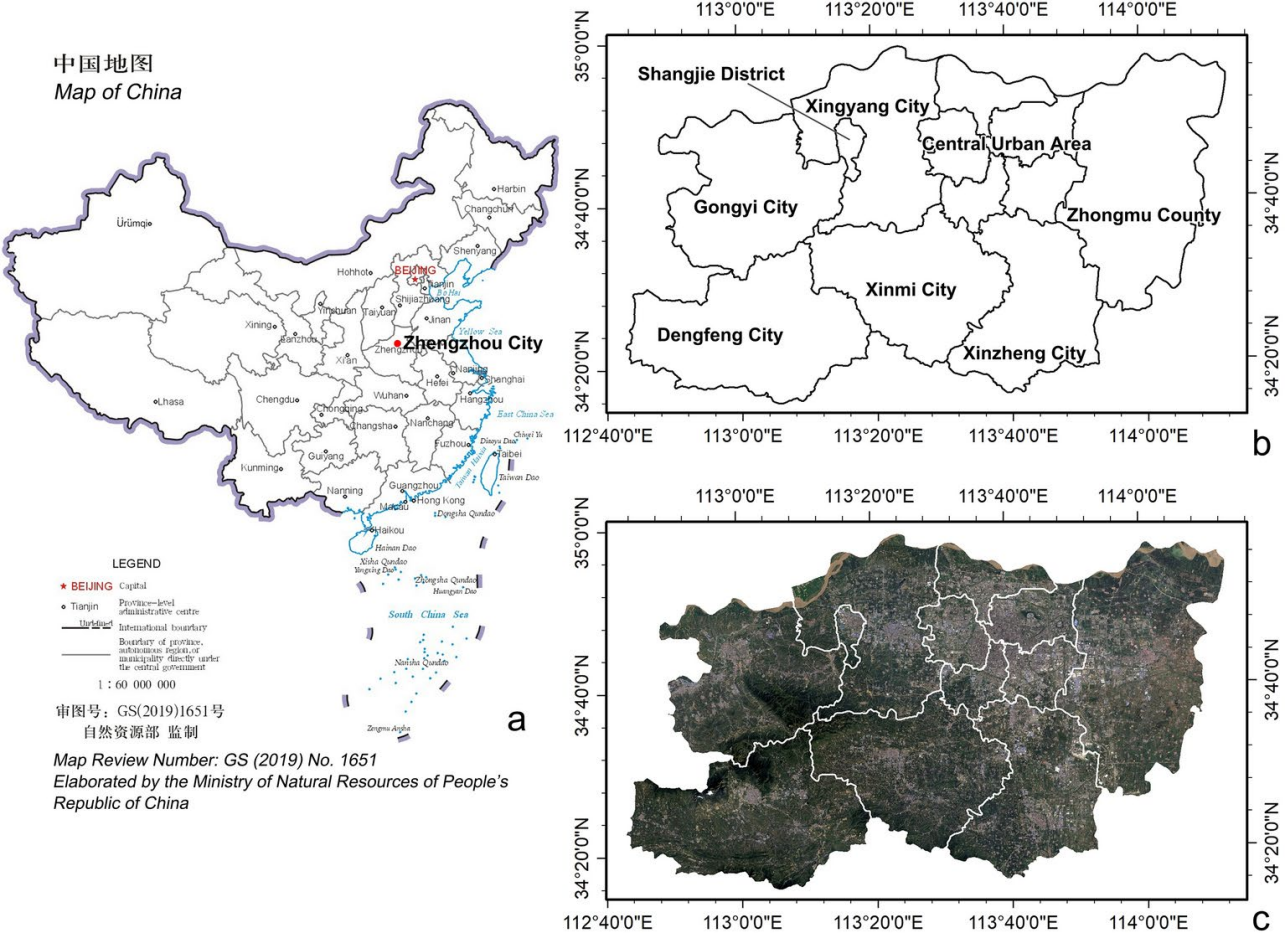
Location and administrative boundaries of the study area: (**a**) location of Zhengzhou City in China, sourced from the Standard Map Service website of the Ministry of Natural Resources of People’s Republic of China (http://bzdt.ch.mnr.gov.cn/). (**b**): administrative divisions of Zhengzhou City, modified from the figure on the Zhengzhou Government website (https://www.zhengzhou.gov.cn/view/index.jhtml). (**c**) the Landsat 8 remote sensing imagery of Zhengzhou City on August 26, 2020, was downloaded from the EarthExplorer website (https://earthexplorer.usgs.gov). Maps (**b**) and (**c**) were elaborated using ArcGIS 10.8.1 (https://www.esri.com/arcgis-blog/products/arcgis-enterprise/announcements/arcgis-enterprise-10-8-1/).

Zhengzhou City is the capital city of Henan Province and one of the significant central cities in the central region of China^37^. It encompasses six districts, five county-level cities, and one county, covering an area of 7567 km^2^. The Central Urban Area includes five districts, forming the primary centre of Zhengzhou City. The county-level cities include Xingyang City, Gongyi City, Dengfeng City, Xinmi City, and Xinzheng City. Shangjie District is located within Xingyang City, and references to Xingyang City in the following include Shangjie District. Zhongmu County is the only county in Zhengzhou City. The topography of Zhengzhou City is relatively complex, with a general trend of being higher in the southwest and lower in the northeast. Gongyi City, Dengfeng City, Xinmi City, and the southern part of Xingyang City feature mountainous and hilly terrain. The Central Urban Area, Xinzheng City, Zhongmu County, and the northern part of Xingyang City are characterised by plain landscapes (Fig. 2).

By the end of 2020, the permanent population of Zhengzhou City was approximately 12.6 million, with urban residents constituting 78.4%^38^. Since 2015, Zhengzhou’s resident population has met the criteria for a megacity. Furthermore, as a national transportation hub, Zhengzhou City has rapidly developed over the past decades, relying on its extensive transportation network^39^. The influx of investment and population has led to the continuous expansion of urban areas^40^. Concurrently, with the support of policies and the drive from township enterprises, certain rural areas have experienced swift de-agriculturalisation and urbanisation^37^. These factors have provided Zhengzhou City with multiple drivers for the formation and development of PUAs. Therefore, choosing Zhengzhou City as a case study for identifying PUAs in megacities is particularly representative.

### Construction of the multi-indicator system

Building on existing research, this study employs multi-source data to establish an indicator system based on four dimensions: urbanisation level, economy, land use, and population (Table 1). These indicators are expected to show significant value differences between urban and rural areas, with the indicator values for PUAs falling between those of these two areas^19,28,32,41^. To enhance the universality of this indicator system, we prioritised selecting data sources that encompass global datasets. The urbanisation level is reflected through Imperviousness Density (ID), an indicator that has been used to identify PUAs in Beijing City^30^ and Wuhan City^26^. Impervious surfaces are areas where land is covered by buildings, roads, or other construction projects, leading to soil sealing^42^. This data is sourced from the 2020 Global 30 m Resolution Impervious Surface Dataset^43^. Nighttime Light Intensity (NLI) reflects the economic level of a region and is also a key indicator for differentiating urban, peri-urban, and rural areas^22,32^. This data is derived from a global NPP-VIIRS-like nighttime light dataset obtained through cross-sensor calibration, with a spatial resolution of approximately 500 m^44^. Proportion of Agricultural land, Forest land and Grassland (PAFG) reflects the degree of naturalisation in a region and exhibits significant differences between urban and rural areas^24^. This indicator is calculated using China’s multi-period land use and land cover remote sensing monitoring dataset, which has a resolution of 30 m^45^. The dataset contains six types of land: agricultural land, forest land, grassland, water, built-up land, and unused land. Per Capita Land Area (PCLA) is primarily calculated using population data, including the census data from the National Bureau of Statistics of China^46^ and the LandScan HD population dataset^47^. The census data is statistical data organised by administrative districts, and we used it in the Threshold method. The LandScan HD population dataset, with a 1 km grid as its statistical unit, was used for the Breakpoint Clustering and Multilayer Perceptron.

**Table 1:** Multi-indicator system for identifying PUAs.

Indicator	Spatialisation	Indicator direction	Weight (Threshold method)	Weight (Breakpoint Clustering)
Imperviousness Density (ID)	The proportion of impervious surface in each unit to the total area of the unit	Positive	0.1460	0.2393
Nighttime Light Intensity (NLI)	The mean value of the night light intensity within each unit	Positive	0.3171	0.1365
Proportion of Agricultural land, Forest land and Grassland (PAFG)	The proportion of the total area of agricultural land, forest land, and grassland in each unit relative to the total unit area of the unit	Negative	0.1244	0.1871
Per Capita Land Area (PCLA)	The value obtained by dividing the average population in each unit by the total area of the unit	Negative	0.4125	0.4370

**Fig. 3:**
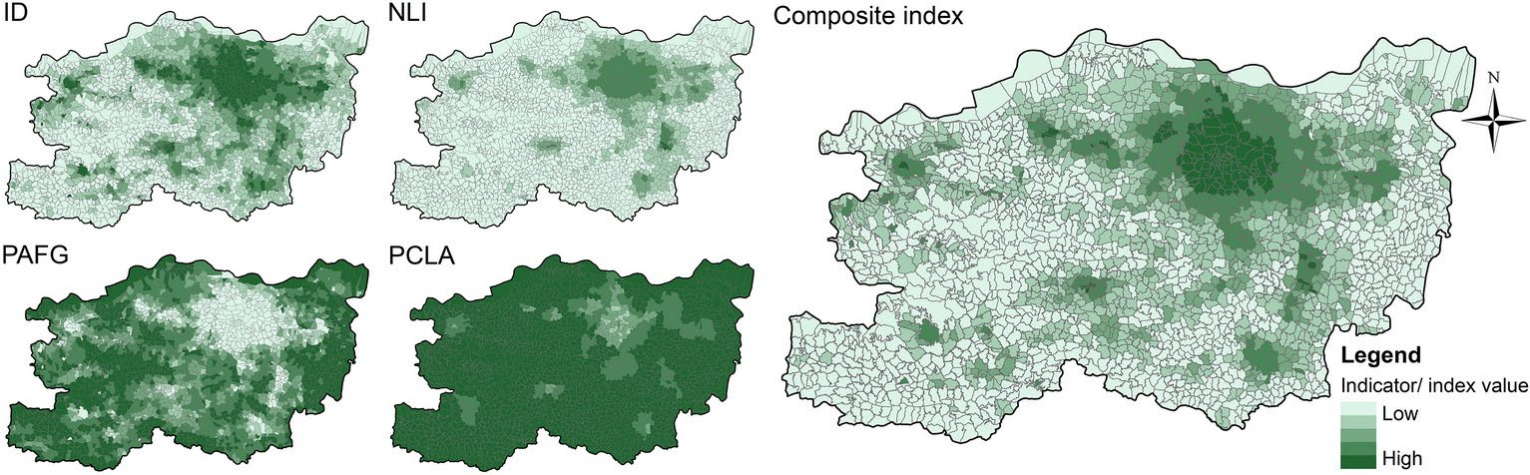
The indicator and composite index values within administrative units, elaborated using ArcGIS 10.8.1 (https://www.esri.com/arcgis-blog/products/arcgis-enterprise/announcements/arcgis-enterprise-10-8-1/).

**Fig. 4:**
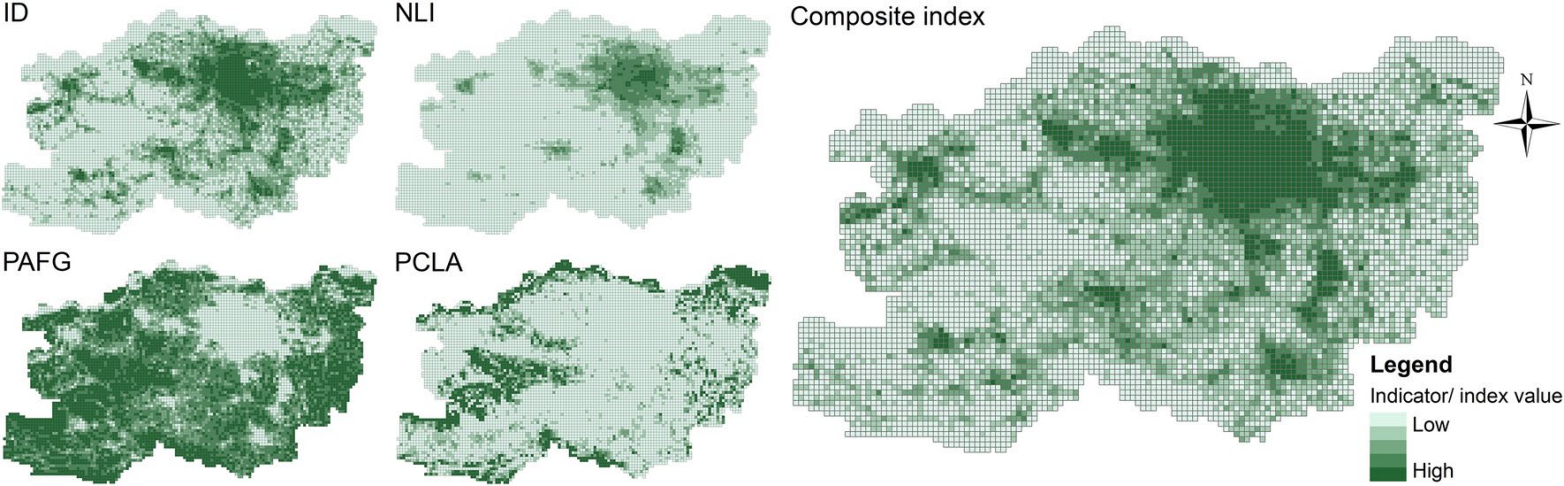
The indicator and composite index values within grid units, elaborated using ArcGIS 10.8.1 (https://www.esri.com/arcgis-blog/products/arcgis-enterprise/announcements/arcgis-enterprise-10-8-1/).

Due to the varying resolutions of the indicator data, it is necessary to perform a unified spatialisation process. This involves partitioning and aggregating all the indicator data using the same research units. The specific calculation methods are detailed in Table 1. The Threshold method uses the smallest administrative units of Zhengzhou City - villages and neighbourhoods - as the research units, totalling 2461. Using the “Zonal Statistics” tool in ArcGIS 10.8.1, the values of the indicators for each administrative unit were calculated (Fig. 3). Given that the smallest statistical unit in the census data is towns and neighbourhoods, not individual villages, we assumed a uniform population distribution within each town, resulting in identical PCLA values for villages within the same town. This assumption affects the precision of the PCLA in rural areas. The Breakpoint Clustering and Multilayer Perceptron use grids with a side length of 1 km as research units, resulting in a total of 7,898 grids within the study area. Similar to the administrative units, all indicator values were recalculated based on the grid units (Fig. 4).

After obtaining the indicator values for each research unit, we applied the range normalisation method to standardise the data and eliminate dimensional effects. To facilitate subsequent calculations, we aggregated the indicators into a composite index, which was then applied in both the Threshold method and the Breakpoint Clustering. The composite index requires alignment in the directionality of the indicators. Among these indicators, ID and NLI are positive indicators, where higher values denote more pronounced urban characteristics, and lower values reflect rural characteristics. In contrast, PAFG and PCLA are negative indicators, where higher values suggest rural characteristics. To ensure consistency, we reversed the direction of the negative indicators, making all indicator values highest in urban areas and lowest in rural areas. We then calculated the composite index value using the following formula:

1 $$C={\sum }_{i=1}^{n}{w}_{i}{x}_{i}$$

where $C$ represents the value of the composite index, ${w}_{i}$ is the weight of the* i*th indicator, ${x}_{i}$ is the value of the *i*th normalised indicator, and $n$ is the total number of indicators. In this study, we used the entropy method to calculate the objective weights of the indicators^48^. Since the Threshold method and the Breakpoint Clustering use different research units, their weights and complex indices also vary (Table 1, Figs. 3 and 4).

### Identification methods of PUAs

In the identification process of PUAs, we employed three methods based on different principles. The Threshold method classifies the composite index based on thresholds automatically identified through data analysis. The Breakpoint Clustering operates on the assumption that there are distinct breakpoints between urban, peri-urban, and rural areas when viewed from any complete urban–rural gradient. By identifying and clustering these breakpoints, we can delineate the inner boundaries (the boundaries between peri-urban and urban areas) and the outer boundaries (the boundaries between peri-urban and rural areas) of PUAs, thus determining their spatial extent^33^. The Multilayer Perceptron is based on principles of machine learning. It constructs a neural network to perform high-level abstraction and classification of the input indicator data, enabling effective regional delineation^49,50^.

#### Threshold method

In this study, we employed the Jenks natural breaks method to obtain thresholds, which offers greater objectivity and data-driven insight compared to predefined thresholds. This method classifies data into several categories based on the inherent distribution characteristics of the data, minimising within-class variance while maximising between-class variance to achieve a reasonable classification of the data^51^. ArcGIS 10.8.1 provides this classification method, allowing the composite index values of 2,461 administrative units to be divided into high, medium, and low categories. The administrative units with composite index values in the middle category will be identified as PUAs.

#### Breakpoint clustering

**Fig. 5:**
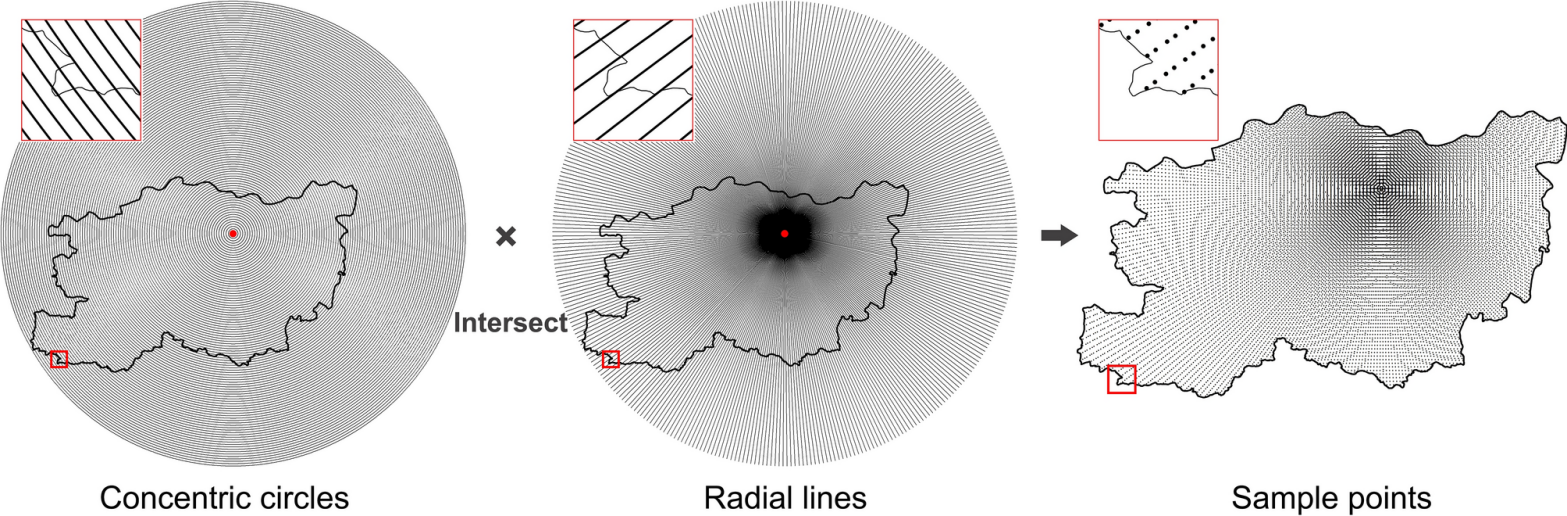
The process of establishing sample points, elaborated using ArcGIS 10.8.1 (https://www.esri.com/arcgis-blog/products/arcgis-enterprise/announcements/arcgis-enterprise-10-8-1/).

To comprehensively simulate the urban–rural gradient, we created a sampling array that radiates outward from the Central Urban Area. The procedure was as follows: the geometric centre of the Central Urban Area (34.77°N, 113.65°E) was used as the focal point to draw 360 radial lines at 1° intervals. Additionally, concentric circles with 1 km intervals were constructed, totalling 97 circles, to cover the entire study area. The intersections of the radial lines and concentric circles yielded 16,334 sample points within the study area (Fig. 5). These sample points were connected to all grid units, allowing each sample point to obtain the composite index value of its corresponding grid unit. In other words, the composite index value of each grid will be linked to one or more sample points.

**Fig. 6:**
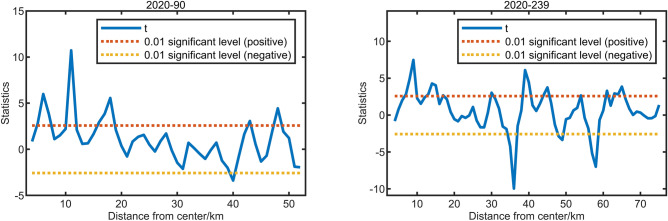
The results of the moving t-test for the 90th and 239th data series.

Next, the sample points were divided into groups based on their radial lines, resulting in 360 data series representing the urban–rural gradients. We conducted moving *t*-tests on these data series using MATLAB R2018a. Moving *t*-test is a statistical method based on examining whether the difference in the means of two sample groups is significant, which allows for the effective identification of breakpoints within data series^19^. Experimental analysis revealed that a test step size of 3 yielded the most accurate and reliable results. As shown in the data series cases in Fig. 6, data peaks exceeding the red line or falling below the yellow line were considered breakpoints. Specifically, data points exceeding the red line indicate a downward shift in the composite index values, while those below the yellow line indicate an upward shift. To ensure the accuracy of the findings, we further cross-validated these breakpoints using remote sensing imagery and removed outliers.

**Fig. 7:**
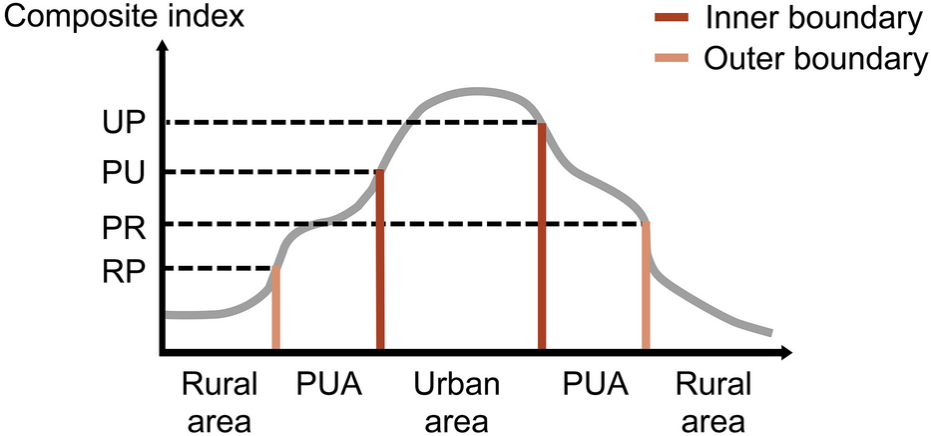
The boundaries and breakpoints of PUA.

Following the classification of breakpoints with upward and downward shifts, we imported these categories into QGIS to perform K-means clustering using the “Clustering” tool. As an unsupervised clustering algorithm, the primary function of the K-means algorithm is to automatically group similar samples into a collection^22^. The breakpoints with upward shifts were observed to fall into two categories: those located at the outer boundaries and those at the inner boundaries. K-means clustering facilitated the differentiation between these scenarios. Breakpoints with higher composite index values were considered situated at the inner boundaries, while those with lower values were deemed to be at the outer boundaries. A similar approach was employed for breakpoints with downward shifts to determine the inner and outer boundaries. Consequently, the breakpoints were categorised into four types: Urban/Peri-urban breakpoints (UP), Peri-urban/Rural breakpoints (PR), Rural/Peri-urban breakpoints (RP), and Peri-urban/Urban breakpoints (PU) (Fig. 7). Additionally, these breakpoints may also be located on the boundaries between urban and rural areas, which we did not specifically list for analysis, but we took note of them in subsequent identification processes. Upon mapping these breakpoints to the corresponding grid units, we assessed other grid units based on these classifications to delineate PUAs.

#### Multilayer perceptron

The Multilayer Perceptron consists of the input layer, hidden layer, and output layer^52^. The input layer receives preprocessed data and relays input vectors to the hidden layers, which are responsible for feature extraction. The output layer presents the model’s final processing results of the input data. Each layer in the Multilayer Perceptron comprises one or more artificial neurons, mimicking the neurons in the brain’s neural network.

**Fig. 8:**
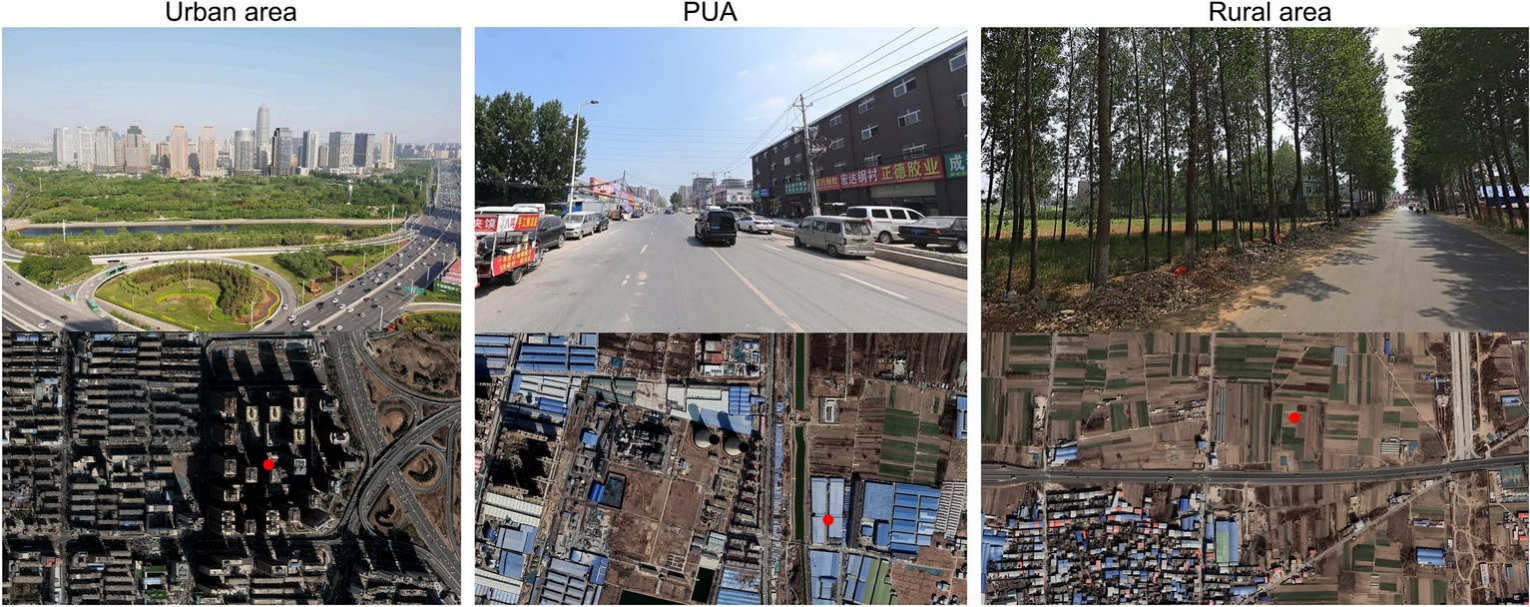
Examples of sample points in urban, peri-urban, and rural areas. The red points represent the sample points, with the remote sensing imagery associated with the red points sourced from the EarthExplorer website (https://earthexplorer.usgs.gov). The corresponding street view images were sourced from the authors.

This study initially employed the “Polygon-to-point” function in ArcGIS 10.8.1 to extract central points from grid units as experimental points, totalling 7898. Each point carries four indicator values. Subsequently, samples were selected based on distinct characteristics of urban, peri-urban, and rural areas for model training. A total of 500 sample points were chosen based on Landsat 8 remote sensing imagery combined with field surveys: 180 urban, 50 peri-urban, and 270 rural (Fig. 8). The sample points were divided into 80% training data, 10% validation data and 10% test data, using a random selection approach.

Subsequently, a Multilayer Perceptron framework was established using PyTorch, a deep learning platform developed by Facebook. The standardised and normalised values of the four indicators from the sample points were used as input layer neurons. A hidden layer comprising 64 neurons was constructed through training and evaluation, utilising the ReLU activation function, which closely approximates human cognitive processes. The ReLU function is defined as:

2 $$ReLU\left(x\right)=\text{max}(0, x)$$

where $x$ represents the input value^53^. To ensure the rationality of the output data, the Softmax function, commonly used in multicategory tasks, was selected as the activation function for the output layer. The function is defined as:

3 $$Softmax\left({z}_{i}\right)=\frac{{e}^{{z}_{i}}}{{\sum }_{j=1}^{K}{e}^{{z}_{j}}}$$

where ${z}_{i}$ represents the input value for each neuron in the output layer, and $K$ denotes the number of categories. Given that the output layer is divided into three categories: urban, peri-urban, and rural areas, $K$ is set to 3. The variable $j$ iterates over all category values of ${e}^{{z}_{j}}$ during the normalisation process, ensuring that the sum of output probabilities equals 1^54^.

The model was trained using the training data with a learning rate set to 0.001 and allowed to iterate for up to 1000 epochs. The Cross-Entropy Loss function, commonly used in neural network classification problems, particularly in multi-class classification tasks, was employed. AdamW was used as the optimiser. It combines the principles of the Adam optimiser with weight decay, effectively mitigating the risk of model overfitting. The model was trained until the global error decreased to 0.001, which served as the convergence criterion to halt the training process. Subsequently, the model was assessed using validation data to identify the optimal model configuration. The model was then assessed using the test data, and it was saved and applied to the fitting of all experimental points only if it met the predetermined accuracy requirements. Finally, the identification of urban, peri-urban, and rural areas was achieved by correlating experimental points with grid units.

### Kappa coefficient

After obtaining the identification results from the three methods, we calculated the kappa coefficients to assess their accuracy. The kappa coefficient quantifies the consistency between the results and the actual conditions by establishing a confusion matrix^9^. We randomly selected 900 sample points within the study area and manually classified them with the assistance of remote sensing imagery and field surveys to simulate actual conditions. The sample points included 200 urban points, 180 peri-urban points, and 520 rural points. Subsequently, we compared the manually classified results of these points with the results from the three methods using the “Compute Confusion Matrix” tool in ArcGIS 10.8.1, which yielded kappa coefficients for each method.

## Results

### Comparison of identification results

**Fig. 9:**
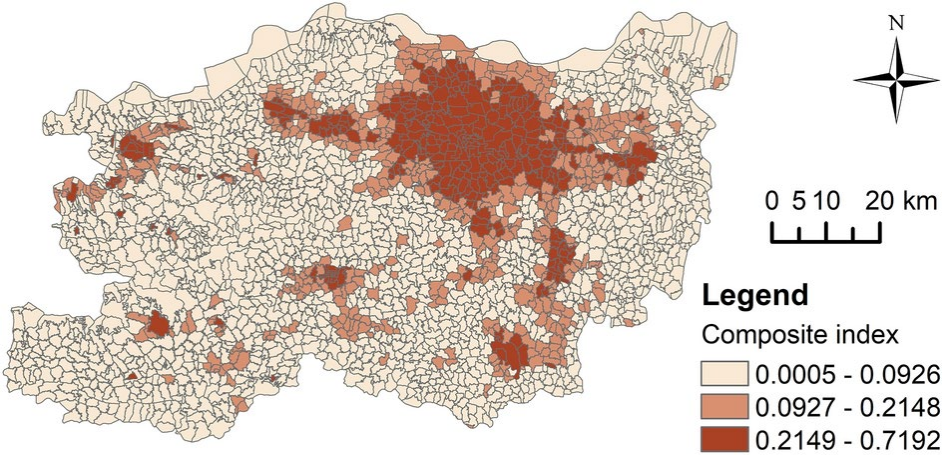
Identification result of the Threshold method, elaborated using ArcGIS 10.8.1 (https://www.esri.com/arcgis-blog/products/arcgis-enterprise/announcements/arcgis-enterprise-10-8-1/).

**Fig. 10:**
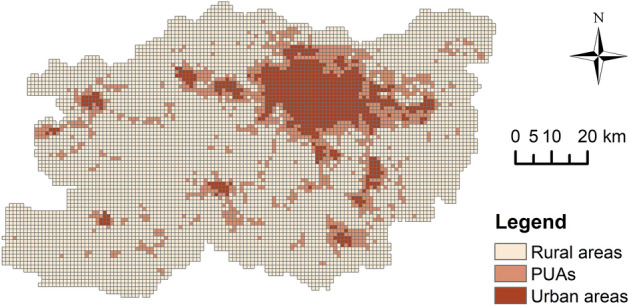
Identification result of the Breakpoint Clustering, elaborated using ArcGIS 10.8.1 (https://www.esri.com/arcgis-blog/products/arcgis-enterprise/announcements/arcgis-enterprise-10-8-1/).

**Fig. 11:**
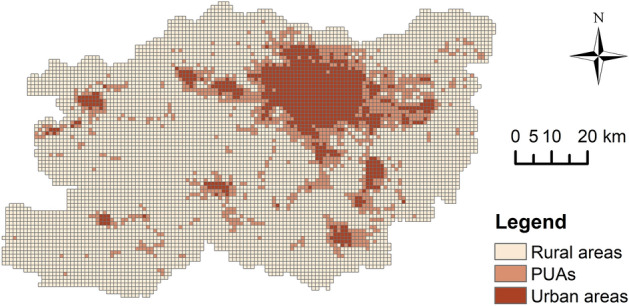
Identification result of the Multilayer Perceptron, elaborated using ArcGIS 10.8.1 (https://www.esri.com/arcgis-blog/products/arcgis-enterprise/announcements/arcgis-enterprise-10-8-1/).

This study obtains three identification results of PUAs from the above methods. Figure 9 shows the results from the Threshold method. The threshold range for the composite index values in PUAs ranges from 0.0927 to 0.2148. The PUAs encompass 468 administrative units, covering an area of 1215.15 km^2^, which accounts for 16.06% of Zhengzhou’s total area. The Breakpoint Clustering identified 2251 breakpoints, comprising 1702 breakpoints with downward shifts (719 UP and 983 PR) and 549 breakpoints with upward shifts (186 PU and 363 RP). Based on these points, the boundaries of the PUAs were delineated (Fig. 10), covering an area of 1138.30 km^2^, representing 15.05% of the total area of Zhengzhou City. The PUAs identified by the Multilayer Perceptron span 1070.31 km^2^, making up 14.15% of Zhengzhou’s total area (Fig. 11).

The similarities and differences in the identification results of the three methods can be observed in Table 2. These results collectively demonstrate that urban areas have the smallest area, while rural areas have the largest. The area of PUAs is slightly larger than that of urban areas but significantly smaller than that of rural areas. For PUAs, the Threshold method identified the largest area, while the Multilayer Perceptron identified the smallest area. Conversely, the situation was entirely opposite in rural areas, with Multilayer Perceptron results closely resembling those of the Breakpoint Clustering. For urban areas, the Threshold method identified the largest area, followed by Multilayer Perceptron, with the Breakpoint Clustering identifying the smallest area.

**Table 2:** The areas of the three types of regions identified by the three methods.

Method	Urban areas (km^2^)	PUAs (km^2^)	Rural areas (km^2^)
Threshold method	890.96	1215.15	5457.29
Breakpoint Clustering	719.00	1138.30	6038.00
Multilayer Perceptron	773.00	1070.31	6051.00

**Fig. 12:**
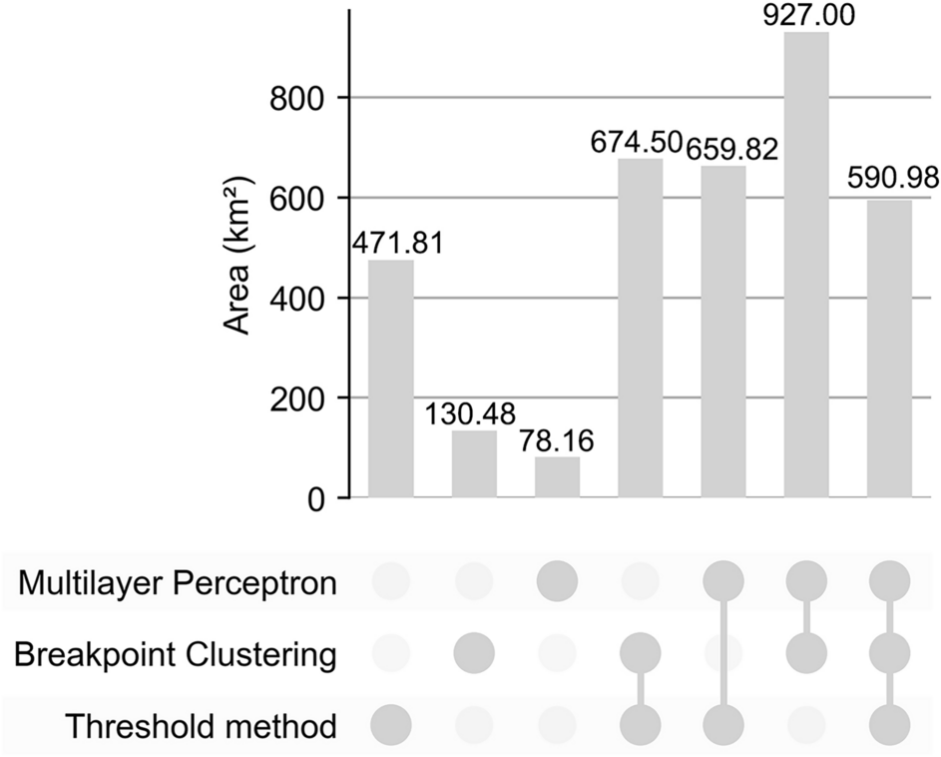
Overlapping areas of PUAs identified by three methods. The bar chart displays the overlapping areas of PUAs identified by the methods corresponding to the deep grey circles at the bottom.

The kappa coefficient for the Threshold method was 0.8504, for the Breakpoint Clustering it was 0.9149, and Multilayer Perceptron achieved the highest kappa coefficient at 0.9422. Further analysis using the “Intersect” tool in ArcGIS 10.8.1 revealed that the identification results of PUAs by the Breakpoint Clustering and Multilayer Perceptron exhibited higher overlap, indicating greater consistency. The overlapping area of PUAs identified by all methods amounted to 590.98 km^2^, which accounts for approximately half of the area of PUAs in each result. In the non-overlapping areas, the area of non-overlapping PUAs identified by the Threshold method significantly exceeds that identified by the other methods, suggesting substantial differences between this result and the other two (Fig. 12).

**Fig. 13:**
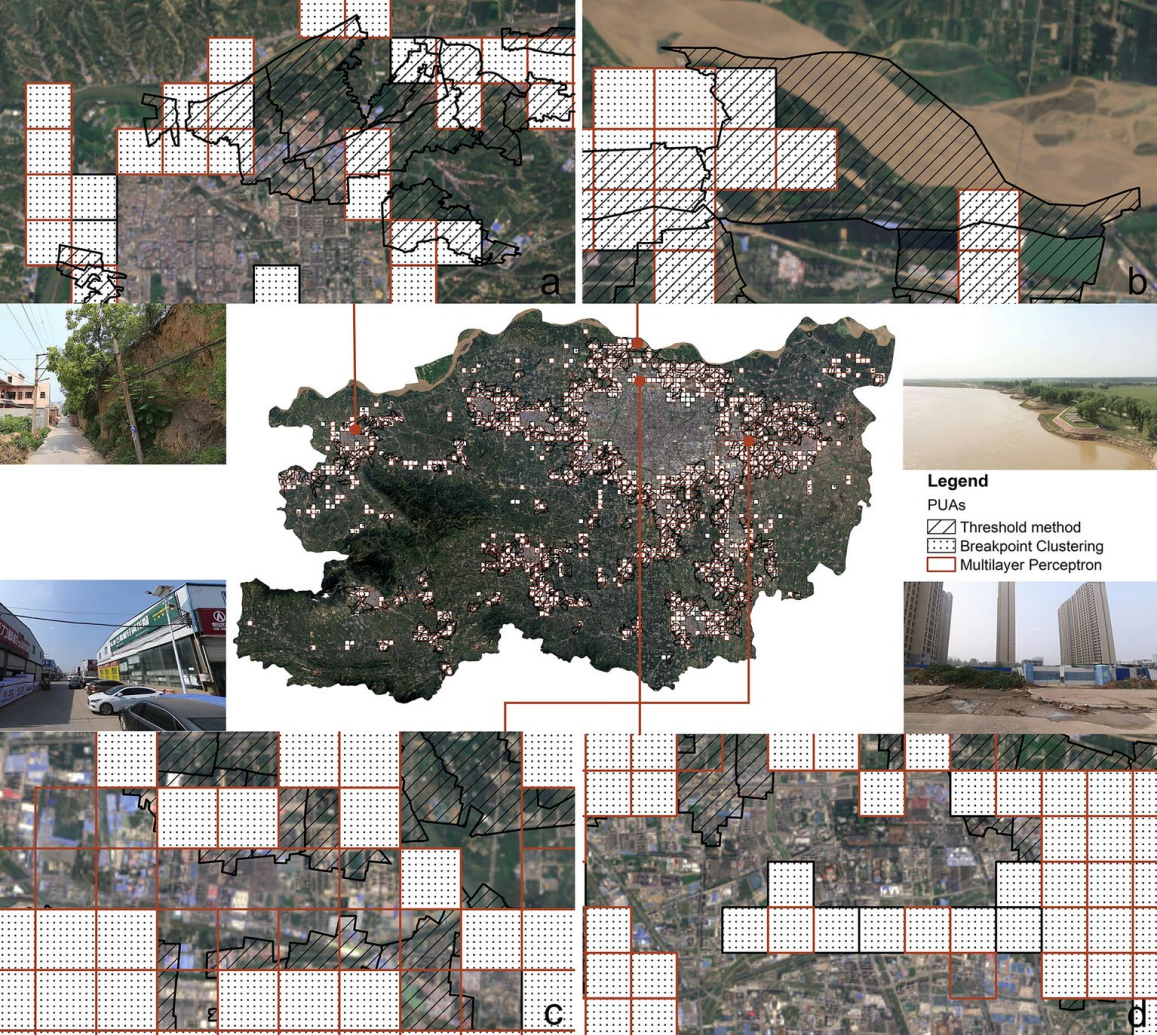
Examples of the discrepancies among the identification results. Remote sensing images were downloaded from the EarthExplorer website (https://earthexplorer.usgs.gov), they were clipped and produced using ArcGIS 10.8.1 (https://www.esri.com/arcgis-blog/products/arcgis-enterprise/announcements/arcgis-enterprise-10-8-1/). Street view images were sourced from the authors.

We then compared the differences between these results by visual interpretation. Figure 13 displays some areas with significant discrepancies. As shown in Fig. 13a, in certain areas of Gongyi City, there is a ring of PUAs surrounding the urban area, with the Threshold method identifying a larger range of PUAs. This is because the administrative units in these areas are quite large, encompassing not only areas with clear urbanisation trends but also parts of agricultural land. Therefore, during the identification process, these agricultural areas were also included in the scope of PUAs. Similar situations occurred in several other cities and county. In contrast, the Breakpoint Clustering and Multilayer Perceptron exhibit smaller errors in this aspect. Figure 13b shows that certain water bodies in the northern part of the Central Urban Area were identified as PUAs by the Threshold method, whereas the other two methods classified them as rural areas. This discrepancy is also related to the larger area of the administrative units used in the Threshold method. Figure 13c represents an area in the eastern part of the Central Urban Area, identified as urban areas by the Breakpoint Clustering and as PUAs by Multilayer Perceptron. Through remote sensing imagery and street view images, it can be observed that there are many blue patches in this region, which correspond to factory buildings and shelters. Additionally, there are scattered farmlands around. In terms of characteristics, these regions tend to be in a transitional state from rural to urban areas, influenced by the radiative effect of the Central Urban Area. Therefore, identifying these areas as PUAs is more reasonable. In the breakpoint Clustering, their classification as urban areas may be due to the relatively low PCLA and high ID in the region, as these two indicators carry the highest weights. Figure 13d shows the new urban expansion areas in the northern part of the Central Urban Area. Although these regions have been designated for urban use, construction has not yet been completed, leading to significant discrepancies in the indicators. For example, the ID is high, while the NLI is low. This also contributes to the differences in classification results in this area.

### Spatial layout of PUAs

The PUAs identified by the three methods exhibit similar spatial layouts. By counting the area of PUAs in the cities and county, we can visually observe the spatial disparities of peri-urbanisation phenomena in Zhengzhou City (Table 3). All results demonstrate that the Central Urban Area has the largest area of PUAs, which are distributed along its periphery. The PUAs in Xinzheng City and Zhongmu County also exhibit significant areas. The areas of PUAs in Xinmi City and Yingyang City are moderate. These four cities are all adjacent to the Central Urban Area. Both Xingyang City and Zhongmu County have flat terrain, while Xinmi City and Xinzheng City feature some mountainous and hilly areas. The PUAs of Gongyi City and Dengfeng City are the smallest. They are located to the west of Zhengzhou City, farther away from the Central Urban Area, and are characterised by mountainous terrain.

**Table 3:** The areas of PUAs in the cities and county of Zhengzhou City.

Method	Area of PUAs (km^2^)						
	Central Urban Area	Xingyang City	Gongyi City	Dengfeng City	Xinmi City	Xinzheng City	Zhongmu County
Threshold method	294.17	134.85	91.31	76.90	153.11	233.61	231.20
Breakpoint Clustering	300.84	117.92	129.10	60.00	123.18	173.62	233.64
Multilayer Perceptron	270.68	114.10	92.45	59.00	111.17	177.92	244.99

From the perspective of spatial morphology, we identified three distinct forms. A large number of PUAs are distributed in a ring form around urban areas, which is most evident in the Central Urban Area. These PUAs can be considered products of urban radiative influence. They are situated between urban and rural areas, serving as a bridge for urban-rural transition. Some PUAs are located in belt-like distributions between closely situated urban areas and can be found between the Central Urban Area and its adjacent cities and county. These PUAs are predominantly formed along roads, facilitated by the radiative influence of multiple urban areas, thereby demonstrating a trend towards networked expansion. Concurrently, these PUAs also fulfil the function of connecting urban areas. A smaller portion of PUAs are distributed in rural areas of various regions in the form of small patches, primarily driven by the intrinsic forces of the rural areas themselves. Due to their smaller size, they do not yet serve the connective function and can only be considered as potential areas for PUAs, awaiting further development and expansion (Figs. 9, 10 and 11).

## Discussion

Previous studies have documented the significance of spatial identification of PUAs; Mortoja and Yigitcanlar (2022), for example, provides evidence from the case of the Brisbane metropolitan area in Australia regarding the consequences of unclear boundaries for PUAs, including misalignment in regional planning, the transition of green spaces to urban land, and obstacles to sustainable development^55^. Cattivelli (2021) introduced legislation and planning documents related to PUAs in Italy, but analysis revealed that the lack of specific spatial delineation for PUAs hindered the effective implementation of these laws and plans^56^. However, there is currently a lack of comparative studies that visually contrast various PUA identification methods, which could serve as a reference for researchers when selecting the most suitable method. In this study, we provided detailed examples of quantitative identification methods for PUAs. The use of the same study area and indicator system allows for comparability among the PUA identification results obtained through different methods, leading to intuitive comparative outcomes. Additionally, we further evaluated these methods from the perspectives of research units, operational processes, and applications. The spatial layout of PUAs also exhibits differences in area and morphology across various regions. By exploring the driving factors that promote the spatial patterns of PUAs, we can predict their future development trends and plan and manage accordingly to guide the sustainable development of PUAs.

### Evaluation of identification methods

Firstly, regarding the selection of research units, the Threshold method employs units whose area is not fixed, contrasting with the fixed-size units used by the other two methods. Among the 2,461 administrative units, only 217 units are smaller than 1 km^2^. The largest administrative unit even reaches 42.25 km^2^. This inconsistency in the size of units could impact the accuracy of indicator calculations, thereby reducing the precision of results to some extent.

Secondly, in terms of the operational process, the complexity of the Breakpoint Clustering and Multilayer Perceptron is significantly higher than that of the Threshold method. The Breakpoint Clustering involves multiple steps and tools, primarily relying on statistical theories to conduct detailed data analysis, which is time-consuming. Multilayer Perceptron requires high accuracy for sample points and often necessitates the integration of various methods, such as remote sensing imagery and field surveys for sample selection. Additionally, it demands substantial training data and expertise in machine learning, which could become a barrier to its widespread adoption. In contrast, the Threshold method is the simplest in terms of operational steps and can directly utilise existing government data.

On the application level, the Threshold method is widely used in studies concerning PUAs. Many studies directly consider specific administrative regions as PUAs for investigation^27,28^. However, some studies have also indicated that the boundaries of PUAs often extend beyond administrative borders, which means that administrative boundaries do not effectively represent the spatial extent of PUAs^56–58^. In contrast, the Breakpoint Clustering method and Multilayer Perceptron can more accurately capture the complex boundaries of PUAs. From a macro perspective, the PUA identification results obtained using the Threshold method have a broader range and are based on administrative units, making them more conducive to planning and policy formulation. In contrast, the Breakpoint Clustering and Multilayer Perceptron can identify PUAs’ boundaries not only at a macro level but also play a role in refined design and management phases. The Breakpoint Clustering method combines the commonly used breakpoint approach and spatial clustering from previous research and can be applied to identifying PUAs across various scales and regions. The application of machine learning in PUA identification is still in its early stages, and the use of Multilayer Perceptron in this field has not yet become widespread. In the future, exploring the extension of this method to more areas holds potential research value.

### Driving factors behind the spatial patterns of PUAs

From the spatial distribution of PUAs, it is evident that PUAs generated by the radiating influence of urban areas cover a much larger area compared to those generated by rural industrial transformation. The radiative effect of the Central Urban Area is particularly pronounced. Urban expansion promotes the formation of PUAs and guides the gradual transformation of land within PUAs from rural to urban functions, a phenomenon observed in many megacities^35^. This process also induces a series of changes within PUAs, including shifts in population, economy, and social structures. Over time, the expanding ring-shaped PUAs on the outskirts of urban areas are likely to merge with the PUAs on the outskirts of adjacent urban areas, forming continuous patches. This is particularly evident between the Central Urban Area and its neighbouring regions, such as Xingyang City, Xinzheng City, and Zhongmu County. In addition to the PUAs driven by the growth of each area’s own urban centre, some PUAs in these locations have formed under the influence of the Central Urban Area. In Italy’s Lombardy and Emilia-Romagna regions, urbanisation has led to a high degree of integration between metropolitan areas and the centres of small to medium-sized towns, resulting in a multipolar PUA continuum^56^.

Geographical factors influence urban planning, which in turn affects the spatial distribution of PUAs to some extent. Guan et al. (2022) studied green space fragmentation in the urban fringe of Ganjingzi District, Dalian, and found that in areas with hilly and mountainous terrain, the expansion of urban construction land has been relatively slow^59^. Compared to the southwestern region, which is characterised by mountainous and hilly terrain, the flat northeastern part of Zhengzhou City has more PUAs. This phenomenon is closely related to urban planning. Gongyi City, Dengfeng City, Xinmi City, and the southern part of Xinzheng City are rich in natural resources. According to the “Master Plan for Zhengzhou Metropolitan Area (2012-2030)” (MPZMA), these areas are primarily designated for ecological protection and cultural tourism. Consequently, many parts of these cities are classified as no-construction zones. This, to some extent, hinders connectivity between regions and affects the development of PUAs. The areas in the southwestern part of Zhengzhou, including Zhongmu County, the northern part of Xinzheng City, and Xingyang City, feature flat terrain, which is conducive to construction activities. In the MPZMA, these areas have been designated as new urban functional zones to support and enhance the Central Urban Area’s functions in modern industries such as manufacturing and logistics. The favourable geographical conditions and strategic planning create optimal conditions for the development of PUAs in these regions. Taking Xinzheng City as an example, on the one hand, the establishment of the Airport Economy Zone in the eastern part of Xinzheng City has driven the improvement of surrounding infrastructure, leading to the transformation of some agricultural lands into urban built-up lands, around which PUAs have gradually emerged. On the other hand, the university town located at the junction of Xinzheng City and the Central Urban Area has attracted new populations and commerce to the original villages, driving urbanisation and peri-urbanisation development in the area. Coupled with the existing old urban area, these factors have resulted in a polycentric distribution pattern of urban areas in Xinzheng City, with correspondingly larger areas of surrounding PUAs. The phenomenon of peri-urbanisation is also very pronounced in Zhongmu County. With its advantageous geographical location and flat terrain, Zhongmu County has attracted numerous enterprises in recent years. The construction of industrial parks has been proceeding vigorously. Large cultural and tourism projects such as the Fangte Adventure Park and the Film Town have attracted a large number of visitors and shops. These factors have collectively facilitated the emergence of PUAs.

The spatial patterns of PUAs are driven by factors such as urban expansion, geography, and urban planning. Consequently, PUAs can quickly reflect the dynamics of urban development. Focusing on these areas can provide timely feedback for governments and planners, allowing for better control over urban planning, enhanced land use efficiency, and promotion of sustainable development^55^. Although the concept of PUA has not been incorporated into urban planning documents, we can explore the extent to which PUAs are influenced by government planning and protection by comparing the spatial relationship between urban built-up areas and PUAs.

**Fig. 14:**
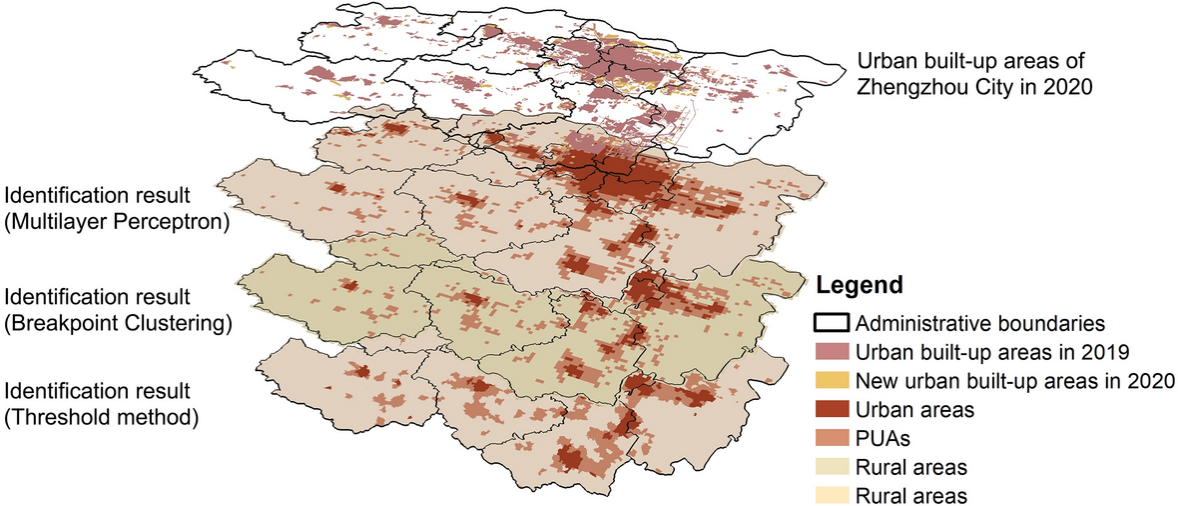
The overlay map of urban built-up areas and the identification results, elaborated using ArcGIS 10.8.1 (https://www.esri.com/arcgis-blog/products/arcgis-enterprise/announcements/arcgis-enterprise-10-8-1/).

In urban planning schemes in China, defining the development boundaries of cities and clarifying land use typically involves delineating urban built-up areas. Urban built-up area specifically refers to non-agricultural land within administrative boundaries that has been requisitioned for construction purposes. It includes concentrated and contiguous portions of urban areas as well as suburban areas with basic and comprehensive municipal infrastructure facilities. Areas designated within urban built-up zones often receive more attention from local governments, which implement detailed planning and management for these regions. In 2020, the urban built-up areas of Zhengzhou City covered an area of 1284.89 km^2^^2,60^. Figure 14 illustrates the overlap between the urban built-up area of Zhengzhou City and the three identification results. It primarily overlaps with the urban areas defined in this study, as well as with some PUAs and a small number of rural areas. By integrating the three identification results, we found that approximately 30% of PUAs are located within the urban built-up areas. This indicates that only a small portion of PUAs is subject to planning and regulation, while nearly 70% of PUAs remain in a state of unregulated development. Figure 14 also shows the newly delineated urban built-up areas in 2020, which are primarily distributed in the eastern part of the study area - a significant region for the contiguous development of PUAs. This phenomenon initially reflects that urban planning and the actual development of PUAs share a similar spatial direction. However, data from just this one year cannot substantiate the evolutionary trend of development directions between PUAs and urban built-up areas. Future analyses can employ data from multiple years to better understand their spatial relationship, thereby clarifying the evolutionary trends of PUAs in relation to planning and regulation.

## Conclusion

This study highlights the importance of accurately identifying PUAs. We found that three quantitative PUA identification methods based on multiple indicators yielded similar results in Zhengzhou City, with the Threshold method showing lower accuracy, while the Breakpoint Clustering and the Multilayer Perceptron demonstrated higher consistency and accuracy. These findings provide researchers with a detailed reference for selecting appropriate PUA identification methods, offering more than just qualitative literature reviews. Additionally, the identification results in our study indicate that a greater number of PUAs are distributed in the northeastern plain area, centred around the Central Urban Area, and they are mainly in the form of rings and belts. This study therefore indicates that the spatial patterns of PUAs may be closely associated with urban expansion, geographical factors, and urban planning. These results also provide valuable insights for policymakers and urban planners to make informed decisions regarding the scientific planning and management of PUAs.

However, some limitations are worth noting. While we have presented three commonly used or novel PUA identification methods, there are still many quantitative identification methods that were not showcased in this study. Additionally, there is a lack of temporal comparison of these methods using multi-year data. The differences in boundary identification between the Breakpoint Clustering and the Multilayer Perceptron also remain to be discussed. These factors contribute to the limitations of the findings in this study. Future research can select a greater variety of PUA identification methods for comparison based on the specific conditions of the study area. Additionally, it should attempt to incorporate more interdisciplinary theories and techniques to guide the precise identification of PUAs. This approach will prepare the groundwork for horizontal cross-regional studies and in-depth longitudinal analyses of PUAs.

## Data Availability

The data generated during and/or analysed during the current study are available from the corresponding author on reasonable request.
